# EU regulation: An unprecedented opportunity to protect children’s and wildlife health from the toxic effects of lead ammunition

**DOI:** 10.1007/s13280-025-02219-4

**Published:** 2025-07-10

**Authors:** Deborah J. Pain, Rhys E. Green, Niels Kanstrup, Rafael Mateo

**Affiliations:** 1 Department of Zoology, University of Cambridge, Cambridge, CB2 3EJ UK; 2 School of Biological Sciences, University of East Anglia, Norwich Research Park, Norwich, Norfolk NR4 7TJ UK; 3 Department of Ecoscience, Aarhus University, C.F. Møllers Allé 8, 8000 Aarhus C, Denmark; 4 Institute for Environmental Assessment and Water Research (IDAEA-CSIC), Jordi Girona 18, 08034 Barcelona, Spain

**Keywords:** EU maximum Pb level, Game meat, Health, Lead ammunition, REACH regulation

## Abstract

Lead is highly toxic to humans and other animals; thus, many uses have been restricted. One exception is lead ammunition, which remains a source of dietary exposure to humans and wildlife. In February 2025, the European Commission published draft regulations for habitat-wide lead ammunition restrictions to protect wildlife and human, particularly children’s, health. These require approval under EU processes. Under EU regulation, Maximum Levels (MLs) for lead have been set for most marketed meats to protect human health, but, surprisingly, no ML has been set for lead in game meat: Here we describe research that supports the urgent need for this. We recommend both approval of the proposed EU ban on lead ammunition use and the setting of MLs for lead in game meat as a complementary measure to aid monitoring and encourage transition to lead-free ammunition globally. These long-overdue measures would protect both children’s and wildlife health.

## Background

Every October, the World Health Organization runs an International Lead Poisoning Prevention Week. In 2024, this aimed to remind “*governments, civil society organizations, health partners, industry and others of the unacceptable risks of lead exposure and the need for action to protect children's health*” (WHO 2024).

Lead (Pb) is a potent neurotoxic metal with no known biological function (ATSDR 2020). Lead products, such as Pb from petrol additives and paint, have been regulated in many countries or globally. Consequently, people’s blood Pb (BPb) levels have declined steadily over the past 40 years, largely in line with decreased atmospheric Pb levels (Angrand et al. 2022). Nonetheless, considerable childhood lead poisoning persists, especially in poor countries where multiple exposure routes exist (Rees and Fuller 2020). Today, most Pb exposure is dietary (EFSA 2010), and one source that has remained largely unregulated is Pb derived from ammunition (bullets and shot) used to hunt wild game animals.

While dietary Pb presents risks to all consumers, it does not affect all consumers equally. Children are especially vulnerable because they absorb a higher proportion of dietary Pb than adults, and the developing nervous system is particularly sensitive to its effects (ATSDR 2020). Pb and calcium (Ca) have a similar ionic size and their divalent cations (Pb^2+^ and Ca^2+^) compete for the same mechanisms of gastrointestinal absorption (Barton et al. 1978; Li et al. 2022). As the efficiency of gastrointestinal Ca uptake is higher in young children and nursing mothers than other groups, this could explain the apparent increase in Pb uptake (COT 2013). The proportion of Pb absorbed may also increase during pregnancy (ATSDR 2020). Neurobehavioral effects in children have been associated with very low BPb levels, and no toxicological threshold has been identified for the effects of Pb on the child’s nervous system (ATSDR 2020). Hence, there is no identified safe BPb level (ATSDR 2020). Effects on cognitive development may be irreversible, and small absolute differences in BPb appear to have proportionately greater effects in the lower part of the range of BPb (< 10 µg/dL) (ATSDR 2020). Therefore, many efforts are being made by health agencies and providers to minimize exposure to Pb of children and pregnant women. Examples include the setting of Blood Lead Reference Values (BLRVs) for children by the U.S. Centers for Disease Control and Prevention with accompanying recommended actions when the BLRV is exceeded (CDC 2025), and the provision of public health advice in the UK highlighting to frequent consumers the importance of cutting down the amount of game meat from animals killed with Pb ammunition eaten, especially by toddlers, children, pregnant women, and women trying for a baby (FSA 2024).

However, to date, few regulatory actions have been taken to protect human health from exposure to ammunition-derived Pb. Those that have been implemented have been largely driven by its impact on wildlife.

### Health risks to wildlife and people exposed to ammunition-derived Pb in game meat: the need for alternative ammunition types

Pb is the main metal used for ammunition globally. In the EU (27), it is estimated that approximately 14,000 metric tons of Pb shot and 134 metric tons of Pb bullets are used annually for hunting (Table 1–10, ECHA 2022a). Most Pb shot fired falls into the environment with a small proportion hitting target game animals, whereas most bullets hit the target animal (Green and Pain 2024). Many waterbirds and terrestrial gamebirds ingest grit which is retained in their muscular gizzard (part of the stomach) to help grind up food. Pb shot ingested mistakenly for grit can be ground up, dissolved by stomach acids and absorbed into the blood stream (Pain et al. 2019). Some species probably also ingest shot mistakenly for seeds of similar size.

Pb shot and bullets that hit the target animal frequently leave many small Pb fragments distributed across the flesh. Game animals that are injured but survive, killed but unretrieved, and the organs and intestines (“gralloch”) of deer and other large game animals that are removed and left in the countryside, provide a source of Pb ammunition and associated Pb fragments that can be ingested by predatory and scavenging animals, primarily birds, including many raptor species (Green et al. 2022a). The conditions in raptors’ stomachs can be very acidic (e.g., Houston 1975); thus, Pb can be readily dissolved and absorbed (Krone 2018). It has been shown experimentally in Bald Eagles (*Haliaeetus leucocephalus*) that while ingested lead shot are often regurgitated, repeated ingestion increases the likelihood of an exposure threshold being reached at which a bird will die from Pb poisoning (Pattee et al. 1981).

Once absorbed into the bloodstream, Pb can result in chronic or acute poisoning of birds. Poisoning of birds exposed to ammunition-derived Pb has been recognized for more than a century (Wetmore 2019) and widely described in the scientific literature (e.g., Pain et al. 2019; Katzner et al. 2024). In Europe alone, in addition to sub-lethal poisoning, Pb poisoning is estimated to kill, annually, over a million wetland birds that directly ingest spent Pb shot (EU 2021), and to additionally reduce the population sizes of some raptor species that eat Pb ammunition fragments in their prey or when scavenging carrion (Green et al. 2022a). There have long been calls to replace Pb ammunition with non-toxic alternatives, principally because of these effects on wildlife. A number of effective alternative (non-toxic) ammunition materials are commercially available. Steel (iron) is the most widely used alternative shot type (Kanstrup and Thomas 2019) and has been in use in some places for more than half a century (Mikula et al. 1977), with copper being the principal component of non-lead bullets (Thomas et al. 2016).

Over the past 20 years, attention has increasingly focused on human health risks. In humans, whole Pb shot or large fragments of Pb ammunition will tend to be removed in the kitchen or at the table or, if accidentally ingested, generally pass rapidly through the alimentary canal. Consequently, health risks from the effects of ammunition-derived dietary Pb in humans tend to be associated with absorption arising from chronic exposure to multiple small fragments of Pb from ammunition. While the shooting of projectiles made from toxic Pb into game meat destined for human consumption might nonetheless appear imprudent, detailed understanding of the nature and extent of associated human health risks has been limited until recently, as described in the next section.

In 2010, the European Food Safety Authority (EFSA) CONTAM Panel produced a Scientific Opinion on Lead in Food (EFSA 2010), which included a specific analysis of risks to adults (but not children) frequently consuming game meat. Various European countries subsequently undertook national risk assessments and consequently advised that the most vulnerable groups (children and pregnant women) should minimize or avoid eating game shot with Pb ammunition. All frequent consumers were advised to butcher game meat carefully to remove damaged meat, and reduce amounts eaten (e.g., BfR 2011; AESAN 2012; Knutsen et al. 2014; ANSES 2018). Key health risks to humans associated with elevated exposure to dietary Pb include increased risk of cardiovascular effects and nephrotoxicity in adults and developmental neurotoxicity in young children (EFSA 2010).

From February 15, 2023, an EU Regulation has prohibited the discharge of Pb gunshot in or within 100 m of wetlands (EU 2021), with the primary aim of protecting waterbirds from Pb poisoning from ingested shot. In 2019, following a report by the European Chemicals Agency (ECHA) on risks to human health and the environment posed by the use of Pb in ammunition in terrestrial environments (ECHA 2018), the European Commission requested ECHA to prepare an additional proposal to restrict the “Placing on the market and use of Pb in projectiles (for firearms and airguns), and in fishing sinkers and lures for outdoor activities.” ECHA’s final dossier proposing restrictions, and the supportive opinion from its risk assessment (RAC) and socio-economic technical committees, were submitted to the European Commission in February 2023 (ECHA 2023). It concluded that the use of Pb in gunshot, bullets, and projectiles poses a risk to wildlife such as birds and to human health that is not adequately controlled and needs to be addressed at the EU level (ECHA 2022a). There was considered to be a high risk to individual birds susceptible to ingesting Pb from ammunition and to populations of some rare bird species (predominantly raptors). With respect to human health, neurodevelopmental effects are the most critical toxicological endpoint of Pb, and there was considered to be a moderate to high risk to those children and pregnant women who frequently eat meat from game hunted with Pb ammunition; it was estimated that a ban of large-caliber lead bullets and lead gunshot could avoid IQ loss in about 7000 children per year (ECHA 2022a). The health risks to other adult groups were considered low (ECHA 2022a, b). In February 2025, a draft regulation proposing the banning of Pb ammunition and fishing weights (with derogations) was presented by the European Commission to representatives in the EU REACH Committee (EU 2025). The final decision on this draft regulation will be taken in comitology (a set of procedures, including meetings of representative committees, that give EU countries a say in the implementing acts) with scrutiny involving the Member States and the European Parliament and Council.

Around the world, evidence of the harm caused by Pb ammunition to wildlife is unequivocal, with much of this available for half a century or more (Wetmore 1919). Evidence of the risks to human health has particularly increased over the past 10–20 years and has added to this. However, this has resulted in limited effective action, with the political and social appetite for managing Pb ammunition varying across stakeholders, administrative regions, countries, and continents (Katzner et al. 2024). Given these impacts and risks, we urge the EU REACH committee of Member States, the European Parliament and Council to fully support the European Commission’s proposal to restrict Pb ammunition.

## Setting maximum levels for Pb in game meat

Food safety standards (Maximum Levels—MLs) for Pb in marketed game meat do not currently exist internationally, despite existing for Pb in most marketed meats, e.g., bovine animals, sheep, pigs, and poultry (hereafter described as farmed animals) (CXS 1995; EU 2023). MLs for Pb have not been set for game meat either at EU level, under the regulation which sets EU Maximum Levels (EUMLs) for certain contaminants, including Pb, in marketed foods (EU 2023), nor within the Codex Alimentarius, (the international "Food Code" adopted within the Joint FAO/WHO Food Standards Programme) “General Standard for Contaminants and Toxins in Food and Feed” (CXS 1995). This is despite the Codex Alimentarius Commission recognizing this source of Pb contamination in its Code of Practice for the Prevention and Reduction of Lead Contamination in Foods (CXC 2004). Taggart et al. (2011) suggested that game meat may historically have been excluded from the legislative framework setting EUMLs for metals (Pb, Cd, and Hg) since the proportion of the population consuming it, and the extent to which it is marketed, may have been limited. These authors also illustrated that these assumptions were not well supported, over a decade ago, by evidence from Europe and North America, and this remains the case.

Both recreational and subsistence hunting are globally widespread. Across Europe, there are an estimated 6 million hunters and 13.8 million individuals in hunter families (3.1% of the EU-27 population; ECHA 2022a). Hunters, their families and friends remain the groups most likely to be high-frequency consumers of game, although a broader range of people are increasingly seeking more naturally-produced foods, such as meat from wild game. Considering only those groups at greatest risks from the health effects of ammunition-derived Pb (ECHA 2022a), European hunters’ families alone include an estimated 2.1 million women of reproductive age, with 130,000 babies born annually, and 1.1 million children of 7 years or younger (ECHA 2022a): These numbers are not trivial. EUMLs for Pb are already set for some other meats from wild species only consumed frequently by a subset of the population, such as cephalopods, crustaceans, and bivalve molluscs (EU 2023).

The EU REACH initiatives described above, and the independent research that they stimulated, have considerably advanced understanding of the risks to human health associated with the consumption of game meat containing ammunition-derived Pb, and provided much information of relevance to the setting of MLs for Pb in game meat.

**Fig. 1:**
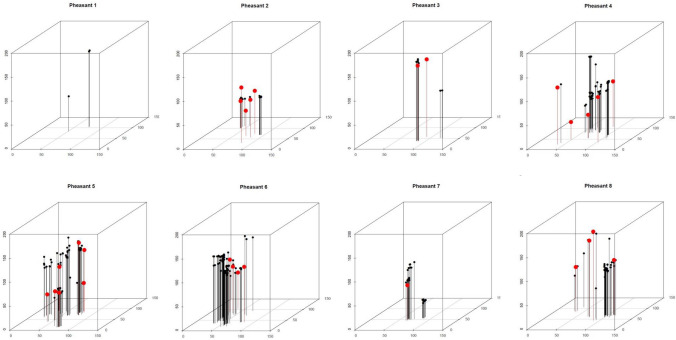
Three-dimensional plots locations of pieces of metal in pheasant carcasses. This shows the locations of metal shards (black circles) and whole or near-whole shotgun pellets (red circles) in the carcasses of eight pheasants. The sizes of the plotted symbols are not to scale. The axes are in millimeters. The grid on the base of each diagram shows 50 × 50 mm squares. Reproduction of Fig. 4 in Green et al. 2022b, PLoS ONE 17(8): e0268089 https://doi.org/10.1371/journal.pone.0268089; CC BY 4.0 http://creativecommons.org/licenses/by/4.0/

First, research has clarified the nature and degree of fragmentation following impact of both Pb bullets, generally used to shoot large game animals like deer, and Pb shotgun ammunition (shot), used to shoot small game animals like birds. While it has long been known that Pb bullets often fragment in game meat leaving behind Pb particles, knowledge of the size and distribution of such particles derived both from bullets and shot is relatively recent and has increased over the past decade. Whole shot or large bullet fragments would be expected to pass through the intestine relatively intact if swallowed. There is less potential for intestinal Pb solubilization from large pieces of Pb-like whole shot with a small surface area/volume ratio, unless these are retained in the intestine, which appears to be rare (e.g., Gustavsson et al. 2005). Experimental work using elemental Pb particles of 6–197 µm in size has shown that a higher proportion of Pb is absorbed from small than large particles in rats (Barltrop and Meek 1979)*.* Recent research shows that Pb bullets and shot often leave numerous Pb particles widely distributed across game meat, some being tens of µm or less in size (Green et al. 2022b; Leontowich et al. 2022). While highly Pb-contaminated meat near the bullet wound channel in large game animals can potentially be removed through careful butchery, some Pb-contaminated meat often remains (Gerofke et al. 2018). When shotgun cartridges are used, multiple individual shot usually hit small game animals, frequently leaving Pb fragments widely distributed across the meat even if all shot have exited the body (Fig. 1; Pain et al 2010; Green et al 2022b). This limits the effectiveness of butchery in reducing exposure. Consequently, fragments of ammunition-derived Pb cannot all be removed and many will be small, thus more readily soluble in the gastrointestinal tract.

Secondly, results from a meta-analysis of Europe-wide studies of Pb in meat from small game animals found that over half of samples had Pb concentrations above the EUML for meat from farmed animals (0.1 mg/kg w.w.) even in studies where all shotgun pellets were carefully removed (Pain et al. 2022). Across studies, variance in mean Pb concentrations decreased substantially as sample sizes increased, with a grand mean (1991–2021; excluding Demark) of c. 2.5 mg Pb/kg w.w., which is 25 times the EUML for Pb in meat from farmed animals. The only country in the dataset with total ban on Pb shotgun ammunition use was Denmark. Here, the mean Pb concentration decreased over time to 0.04 mg/kg w.w. (2011–2021) (Pain et al. 2022), illustrating the effectiveness of the total ban and its potential benefits for human health. As for small game, similarly elevated Pb concentrations have been found in meat from large game animals killed using bullets (Green and Pain 2024).

Thirdly, although it has long been known from dosing experiments with rats that some elemental lead can be absorbed (Barltrop and Meek 1979), relatively little was known of the bioavailability of ingested elemental Pb derived from ammunition. However, correlative studies in humans (Green and Pain 2012) and dogs (Fernández et al. 2021) and an experimental study in pigs (Hunt et al. 2009) showed clear positive associations between consumption of wild-shot game and blood Pb concentrations. These associations appear to be dose-dependent in humans (Green and Pain 2012). In vitro studies also reflect this bioavailability of Pb from ammunition, especially after cooking the meat with acidic recipes (Mateo et al. 2011). In human studies that correlate BPb with levels of game consumption, other types of hunting-associated exposure to Pb, such as through inhalation of Pb fume or dust during shooting, can be a confounding factor. However, this is not true for the studies of dogs and pigs. Collectively, the evidence for absorption of some Pb from ingested ammunition fragments is now compelling.

Another consideration when setting MLs is that Pb fragments from ammunition are unevenly distributed across the meat of individual game animals (Fig. 1); thus, concentrations are highly variable. This contrasts with concentrations of most or all contaminants for which EUMLs have been set as they will result from biologically incorporated (rather than shot-in) exposure and thus be relatively homogenous across an individual tissue type within an animal. Nonetheless, for frequent consumers, who will be exposed to a variety of Pb concentrations in game meat consumed across the year, arithmetic mean Pb concentrations are an appropriate indicator of overall exposure. This is important from a health perspective because it is unknown whether occasional very high exposures or more frequent moderately elevated exposures differ in the harm potentially caused, especially at critical times in a child’s development. Additionally, bone Pb concentrations increase with age, and some bone Pb can be remobilized, helping maintain elevated BPb levels after exposure decreases. Bone Pb in pregnant women can be a source of Pb to the developing fetus (ATSDR 2020). Consequently, occasional very elevated exposures and more frequent moderate exposures to dietary Pb may both elevate health risks.

Potential reasons for not previously setting EU and global MLs for Pb in game meat, should they be related to frequency of consumption, as suggested by Taggart and colleagues (Taggart et al. 2011) and/or other factors mentioned above, do not appear valid. Commission Regulation (EC) No. 1881/2006 on maximum levels for certain contaminants in food (EC 2006) was updated and replaced in 2023 by Commission Regulation (EU) 2023/915 (EU 2023). In this revision, some of the EUMLs for Pb in meat of farmed animals were reduced, including in offal from bovine animals, sheep, pigs, and poultry. We are unaware of why this timely opportunity for the inclusion of an EUML for Pb in game meat was missed, as much of the information cited here would already have been available.

Following the risk assessment process undertaken by ECHA as part of the EU REACH process described above, the ECHA RAC strongly recommended setting an EUML for Pb in game meat, similar to the MLs of Pb for farmed animals, i.e., 0.1 mg/kg w.w. (ECHA 2022b). The Codex Alimentarius General Standard for Contaminants (CXS 1995) has a range of principles regarding contaminants in food and feed, include that: MLs shall only be set for food in which the contaminant may be found in amounts that are significant for the total exposure of the consumer, and that MLs shall be set in such a way that the consumer is adequately protected. Risk assessments illustrate that frequent consumption of wild game meat containing average Pb levels substantially elevates dietary Pb exposure. A UK risk assessment estimated that consumption of two wild-shot game bird meals every week throughout the year would increase the dietary exposure to Pb by up to 8 times for an adult and up to 5 times for a toddler (FSA 2012). In the EU, EFSA estimated a 2.5-fold increase of Pb exposure for frequent consumption of game meat by adults (no estimate was made for children) (EFSA 2010). The comprehensive risk assessment undertaken by ECHA concluded that there was a moderate to high risk to children and pregnant women who frequently eat meat from game animals hunted with Pb ammunition (ECHA 2022a, b). This illustrates the need for setting MLs for Pb in game meat. Pb ammunition is used to kill wild game globally, and there is considerable international trade in wild-shot game meat. Consequently, we recommend that health-protective MLs for Pb in game meat should be set as a matter of urgency at both EU and global levels.

## If use of Pb ammunition for hunting is banned will an EUML still be needed?

Some may argue that EUMLs for Pb in game meat need not be considered, given that most uses of Pb ammunition for hunting might soon be banned in the EU. The draft restriction of Pb ammunition prepared by the European Commission (EU 2025) will be voted on by representatives of the EU Member States on the REACH Committee and scrutinized by the European Parliament and Council before adoption into law, if accepted. This process is partly political and thus subject to many pressures unrelated to scientific evidence. Until such time as the proposal is adopted into law, health risks from Pb in game meat will continue.

Regardless of EU Pb ammunition restrictions, the setting of an EUML for Pb in game meat would be an important standalone health-protective measure. Many European countries are major importers of game meat (FFE 2022). More game is imported to than exported from Europe, and few countries globally have Pb ammunition restrictions, beyond those for shooting waterbirds or in wetlands (Katzner et al. 2024). While certain countries exporting game meat to the EU may adequately monitor residues of Pb in game meat, thus reducing risks of importing Pb-contaminated meat, an EUML for Pb in game would provide an important additional health-protective measure. It would also be a useful complementary measure to ammunition regulation in the EU as regards assessment of compliance with regulations. Compliance with regulations restricting the use of Pb shot use in wetlands and/or for shooting waterbirds in Europe has generally been poor (Pain et al. 2022). In the EU, only Denmark and the Netherlands have total bans across all habitats on Pb shot use for hunting. In 2008, the Danish surveillance program for heavy metals in food, which includes game meat, had for several years identified samples of game, especially pheasants, with Pb concentrations above the national action threshold for poultry (equivalent to the EUML for farmed meats of 0.1 mg/kg w.w.). Research identified limited compliance with the Pb shot ban as the cause (Kanstrup 2012). This resulted in an information campaign, after which compliance improved and Pb concentrations in game meat decreased (Kanstrup 2012; Kanstrup and Balsby 2019; Pain et al. 2022). The setting of an EUML for Pb in game, with associated monitoring, would thus serve the additional function of helping to monitor compliance with Pb ammunition restrictions.

## Time to protect children’s and wildlife health from Pb in game meat

The setting of an EUML for Pb in game meat has previously been recommended by scientists (Pain et al. 2010; Taggart et al. 2011; Thomas et al. 2020) and, more recently, strongly recommended by ECHA’s RAC at a level similar to that already existing for other meats (ECHA 2022b). Liaison between ECHA and EFSA, the authority responsible for EUMLs, should be facilitated by recent EU “One substance, one assessment” chemicals reform (EC 2023) and a Commission-adopted proposal aimed at strengthening cooperation, and consolidating work on chemicals among these and other relevant agencies. As it stands, there is an anomalous mismatch between the position of the EU on MLs (for which EFSA usually advises levels that would result in a rejection rate of less than 5% (AGRINFO 2022)), the recommendation of ECHA RAC that EUMLs should be set (ECHA 2022b), and the advice given by health authorities in several EU states that vulnerable groups of consumers should avoid eating meat from animals killed using Pb ammunition (Knutsen et al. 2014).

Setting an evidence-based health-protective EUML now would probably identify its exceedance by a high proportion of wild game meat marketed in the EU; hence, there may be opposition to its introduction from various groups with vested interests. We argue that this should not impede the setting of standards that protect the health of vulnerable groups, particularly toddlers, children, and pregnant women or those trying for children. Already, the UK food redistribution charity Fareshare does not redistribute wild game to charities where these groups are being fed (Fareshare 2024). We also strongly recommend that additional measures be taken to enhance awareness of caretakers, parents, or guardians of the risks of feeding game meat shot with Pb ammunition to children, and that pregnant women are similarly sensitized to associated risks. This is especially important for those groups most likely to frequently consume wild game, including hunters and their families.

This article has an EU focus because the setting of an EUML for Pb in game meat, and the proposed restriction of Pb ammunition use, are currently topical. However, this issue is of global significance. Inclusion of a ML for Pb in game meat in the Codex Alimentarius international “Food Code” is similarly important and would highlight the need for additional national and/or regional action on MLs. This issue does not affect everyone equally. As described above, firstly children absorb more of a given amount of dietary Pb than adults and, secondly, their developing nervous systems are particularly sensitive to its effects. Moreover, young children generally eat what their parents feed them, thus do not have the option of making an informed choice about consumption, and so suffer triple jeopardy from elevated dietary Pb. Globally, disproportionate exposure to ammunition-derived Pb may occur in subsistence hunting communities that depend upon game meat (Pain and Green 2019), along with workers in the game industry that are routinely given parts of game animals most likely to contain Pb (DFFE 2023).

While we argue that the setting of EU and global MLs for Pb in game meat are both necessary and urgent, approval and implementation of the draft EU regulatory ban on the use of Pb ammunition (EU 2025) is critical, not only for protecting the health of all game meat consumers, but also that of birds, including populations of some of Europe’s most charismatic raptor species (Green et al. 2022a), other, non-avian wildlife (Chiverton et al. 2022), domestic pets (Pain et al. 2023), and farmed animals (Payne et al. 2013).

Solutions to the risks posed by dietary exposure to ammunition-derived Pb to humans and wildlife exist which legislators and regulators need to consider comprehensively and act on without delay. Perhaps by the next WHO International Lead Poisoning Prevention Week in October 2025, there will be regulatory progress to report, at least at European level.
